# Overexpression of carbonic anhydrase IX induces cell motility by activating matrix metalloproteinase-9 in human oral squamous cell carcinoma cells

**DOI:** 10.18632/oncotarget.20236

**Published:** 2017-08-12

**Authors:** Jia-Sin Yang, Chiao-Wen Lin, Yi-Hsien Hsieh, Ming-Hsien Chien, Chun-Yi Chuang, Shun-Fa Yang

**Affiliations:** ^1^ Department of Medical Research, Chung Shan Medical University Hospital, Taichung, Taiwan; ^2^ Institute of Medicine, Chung Shan Medical University, Taichung, Taiwan; ^3^ Institute of Oral Sciences, Chung Shan Medical University, Taichung, Taiwan; ^4^ Department of Dentistry, Chung Shan Medical University Hospital, Taichung, Taiwan; ^5^ Institute of Biochemistry, Microbiology and Immunology, Chung Shan Medical University, Taichung, Taiwan; ^6^ Graduate Institute of Clinical Medicine, Taipei Medical University, Taipei, Taiwan; ^7^ Department of Medical Education and Research, Wan Fang Hospital, Taipei Medical University, Taipei, Taiwan; ^8^ School of Medicine, Chung Shan Medical University, Taichung, Taiwan; ^9^ Department of Otolaryngology, Chung Shan Medical University Hospital, Taichung, Taiwan

**Keywords:** carbonic anhydrase, matrix metalloproteinase, metastasis, migration, OSCC

## Abstract

Oral cancer is a solid malignant tumor that is prone to occur following hypoxia. There are no clear studies showing a link between hypoxia and oral carcinogenesis. Carbonic anhydrase IX (CAIX), which is a hypoxia-induced transmembrane protein, is highly expressed in various types of human cancer. However, the effects of CAIX on the metastasis of human oral cancer cells and the underlying molecular mechanisms have not been clarified. In this study, we observed that CAIX overexpression increased the migratory and invasive abilities of SCC-9 and SAS cells. In addition, CAIX overexpression increased the mRNA and protein expression of matrix metalloproteinase-9 (MMP-9) and the phosphorylation of focal adhesion kinase (FAK), steroid receptor coactivator (Src), and extracellular signal-regulated kinase 1/2 signaling proteins. CAIX overexpression also increased the binding capacity of nuclear factor-κB (NF-κB), c-Jun, and c-Fos on the MMP-9 gene promoter. In addition, treatment with MMP-9 short hairpin RNA, an MMP inhibitor (GM6001), an FAK mutant, or an MEK inhibitor (U0126) inhibited CAIX-induced cell motility in SCC-9 cells. Moreover, data sets from The Cancer Genome Atlas demonstrated that CAIX expression was significantly associated with advanced progression and poor survival in oral cancer. In conclusion, it can be inferred that CAIX overexpression induces MMP-9 gene expression, which consequently induces the metastasis of oral cancer cells.

## INTRODUCTION

Oral cancer is a major subgroup of head and neck cancers and remains a common malignancy worldwide [1]. In Taiwan, oral cancer is the fourth leading cause of cancer death and is associated with increasing morbidity and mortality rates [2, 3]. Despite technological advancement in treatment modalities and diagnosis, the 5-year survival rate of oral cancer patients has not significantly improved during the last half century [4]. Among malignant neoplasms, the overall survival rate of oral cancer patients is relatively low due to metastasis and recurrence [5, 6]. Thus, a comprehensive understanding of the biological nature of this aggressive disease and the development of novel effective therapy for this disease are urgent.

Carbonic anhydrase IX (CAIX) belongs to the α family of CA, which comprises zinc metalloenzymes that catalyze the reversible hydration of carbon dioxide to bicarbonate ions and protons (CO_2_ + H_2_O ↔ HCO_3_^−^ + H^+^) [7]. CAIX is a membrane-associated glycoprotein that consists of signal peptides, a proteoglycan-like domain, CA domains (which have a highly conserved active site), a transmembrane anchor, and an intracytoplasmic tail [8]. CAIX expression is induced by hypoxia through the binding of the hypoxia-inducible factor (HIF)-1 to a hypoxia-response element within the basal promoter of its gene (CA9), subsequently mediating transcription [9, 10]. CAIX supplies bicarbonate ions for the neutralization of intracellular pH and provides protons for the acidification of the extracellular microenvironment, which contribute to promoting cell survival and proliferation, reducing cell adhesion, activating proteases that degrade the extracellular matrix (ECM), and increasing cell migration and invasion [11-14].

In normal human tissues, CAIX expression is limited to the gastrointestinal mucosa, pancreatic ducts, biliary tract and the gallbladder, ovaries, testes, hair follicles, mesothelium, and choroid plexus [15]. However, CAIX is frequently overexpressed in various solid tumors, including oral cancer [13, 14, 16-19]. Eckert et al. reported that a low expression of CAIX combined with a low expression of HIF-1α was significantly correlated with an improved prognosis among patients with oral squamous cell carcinoma (OSCC) [20]. Brockton et al. stated that elevated stromal CAIX expression was associated withnodal metastasis and reduced 5-year disease-specific survival in patients with OSCC [17]. Moreover, our previous studies have shown that higher plasma levels of CAIX were significantly associated with an advanced TNM stage [13], and that elevated CAIX expression in the tissue was associated with an advanced pathological stage, lymph node metastasis, and poor overall survival in Taiwan [14]. These findings showed that high CAIX expressions in clinical specimens from patients with oral cancer are significantly associated with poor outcomes. However, the role of CAIX in oral cancer cells and its molecular mechanism are still unclear. Therefore, in this study, we investigated the function of CAIX in oral cancer cells. Moreover, the signaling pathway and transcription factor in CAIX-overexpressing oral cancer cells were elucidated.

## RESULTS

### CAIX expression was significantly upregulated and associated with advanced progression and poor survival in oral cancer according to the data from the cancer genome atlas

**Figure 1:**
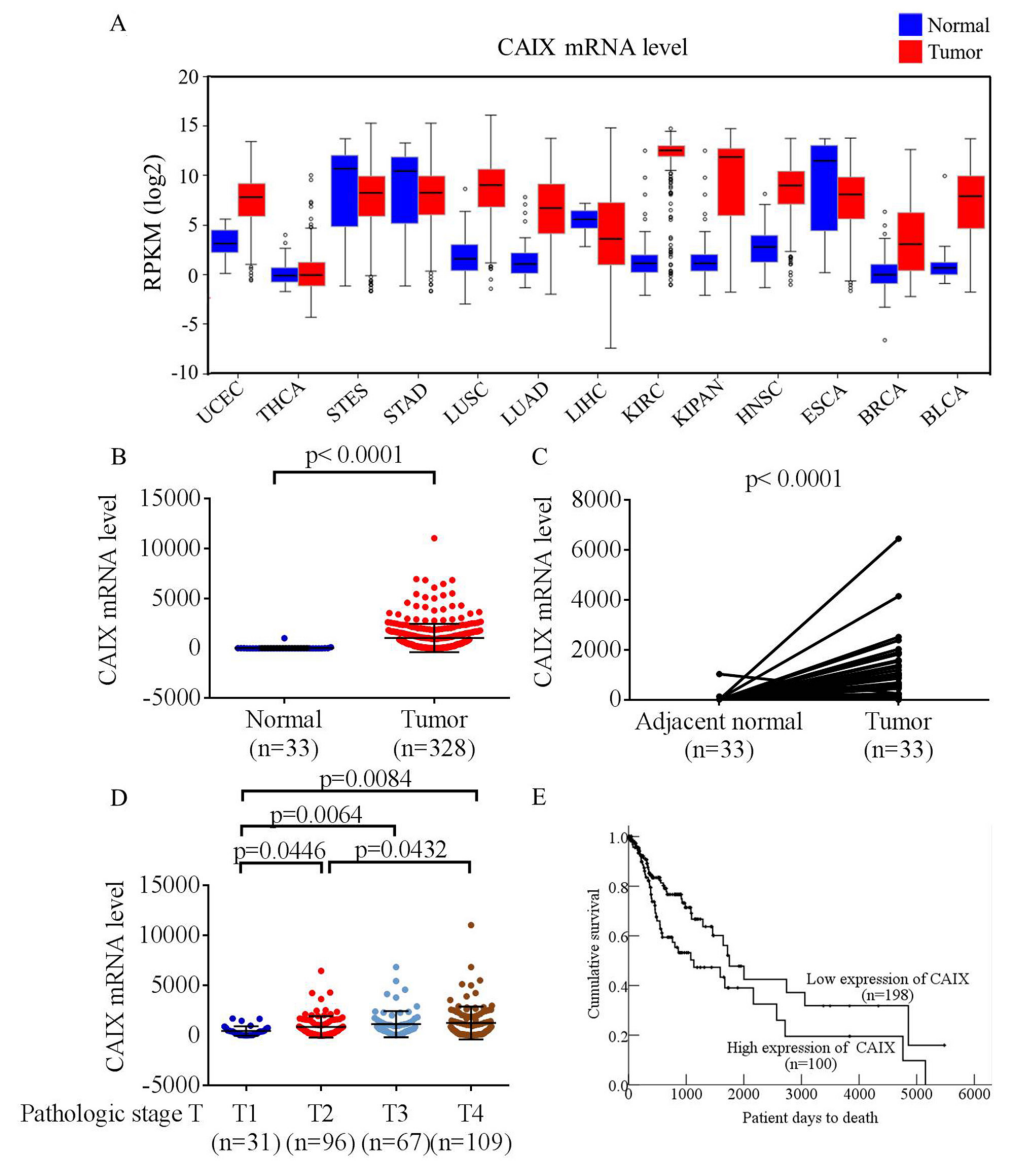
Analysis of CAIX RNA expression in The Cancer Genome Atlas (TCGA) microarray data set **(A)** Analyses of CAIX expression in all types of cancer in TCGA microarray data set. UCEC, uterine corpus endometrial carcinoma; THCA, thyroid carcinoma; STES, stomach and esophageal carcinoma; STAD, stomach adenocarcinoma; LUSC, lung squamous cell carcinoma; LUAD, lung adenocarcinoma; LIHC, liver hepatocellular carcinoma; KIRC, kidney renal clear cell carcinoma; KIPAN, pan-kidney cohort (kidney chromophobe + KIRC + kidney renal papillary cell carcinoma); HNSC, head and neck squamous cell carcinoma; ESCA, esophageal carcinoma; BRCA, breast invasive carcinoma; BLCA, bladder urothelial carcinoma. **(B)** Analyses of CAIX expression in normal oral tissues (n = 33) and oral tumors (n = 328) in TCGA microarray data set. **(C)** Analyses of CAIX expression in 33 pairs of normal and tumor oral tissues in TCGA microarray data set. **(D)** Analyses of CAIX expression in 303 patients with oral cancer in TCGA microarray data set. **(E)** Effect of CAIX expression on the overall survival of 298 patients with oral cancer evaluated using the Kaplan–Meier method.

To explore the clinical value of CAIX in patients with malignant tumors, we analyzed CAIX mRNA expression in data sets from The Cancer Genome Atlas (TCGA) and found that most CAIX transcripts were overexpressed in carcinoma tissues compared with noncancerous tissues (Figure 1A). In addition, the mRNA level of CAIX in patients with oral cancer was significantly higher in tumor tissues than in matched adjacent normal tissues (Figure 1B and 1C). Furthermore, to elucidate the clinical role of CAIX in patients with oral cancer, we analyzed the relationships between CAIX expression and clinical parameters. CAIX expression in patients with oral cancer was augmented in stages T2, T3, and T4 compared with that in stages T1 (Figure 1D). Kaplan–Meier survival analysis revealed that patients with high levels of CAIX (separation by means) exhibited shorter survival than patients with low levels of CAIX (p = 0.011, Figure 1E). This finding indicates that patients with oral cancer with higher levels of CAIX have a poorer prognosis. Moreover, these results suggested that CAIX upregulation is significantly associated with the advanced progression of human oral cancer.

### CAIX-induced oral cancer cell migration and invasion

**Figure 2:**
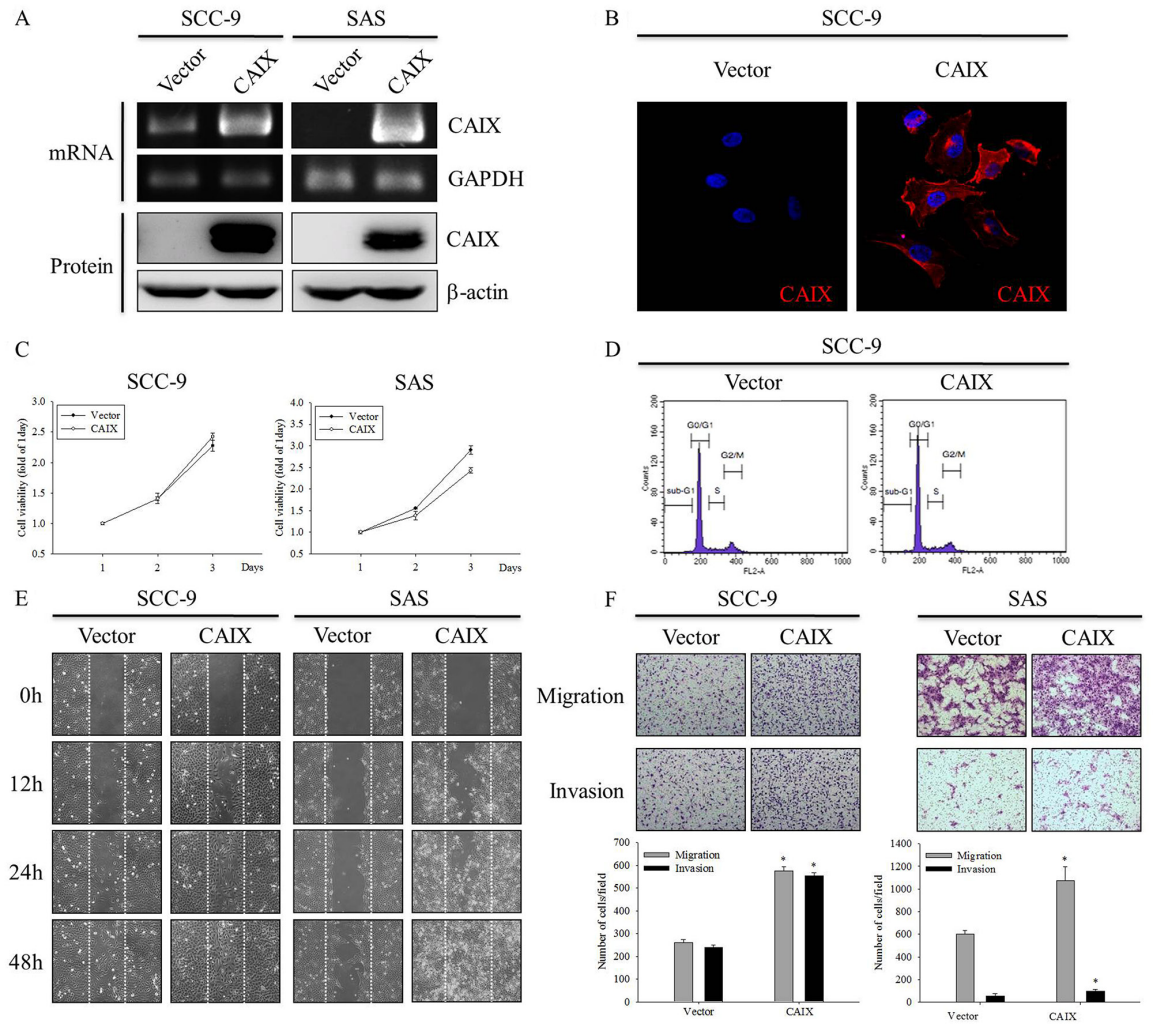
CAIX overexpression induces oral cancer cell motility SCC-9 and SAS cells were stably transfected with an empty vector control or the CAIX expression plasmid. **(A)** (upper) The expression mRNA levels of CAIX in plasmid-transfected SCC-9 and SAS cells were analyzed using RT-PCR. GAPDH was used as a loading control. (lower) Cell lysates were analyzed using Western blotting to detect the expression levels of CAIX. b-actin was used as a loading control. **(B)** The expression of CAIX protein in SCC-9 cells was detected using ICC. **(C)** Growth curves of SCC-9 and SAS cells with or without CAIX expression was examined using the MTT assay. **(D)** Cell cycle profiles of transfected SCC-9 cells were determined using flow cytometry. **(E)** Cell motility was assessed using the wound healing assay. **(F)** The migratory and invasive abilities of SCC-9 and SAS cells were examined using the Boyden chamber assay. (upper) Images were obtained using an inverted contrast light microscope under 100× magnification. (lower) The migratory and invasive abilities were determined by counting the migrated and invaded cells in three randomly selected microscopic fields per well. The results are expressed as mean ± SD of three replications. *Statistically different from vector control at p < 0.05 according to the two-tailed Student’s t-test.

To investigate the role of CAIX in oral cancer cells, we established stable transfectant SCC-9 and SAS cell lines by transfecting these cell lines with CAIX. As shown in Figure 2A and 2B, the expression level of CAIX increased in CAIX-overexpressing SCC-9 and SAS cells, as confirmed by RT-PCR, Western blotting, and ICC analysis. However, the MTT assay and flow cytometry showed that SCC-9 and SAS cells overexpressing CAIX did not exhibit any change in their proliferation rate (Figure 2C) and cell cycle profiles (Figure 2D) when compared with vector control cells. Furthermore, we examined the effect of CAIX on the migratory and invasive abilities of SCC-9 and SAS cells by using the wound healing and Boyden chamber assays. As shown in Figure 2E and 2F, CAIX overexpression significantly promoted the migration and invasion of both SCC-9 and SAS cells. These results demonstrate that CAIX expression plays a critical role in the migratory and invasive potentials of human oral cancer cells.

### CAIX markedly upregulated MMP-9 in SCC-9 cells

**Figure 3:**
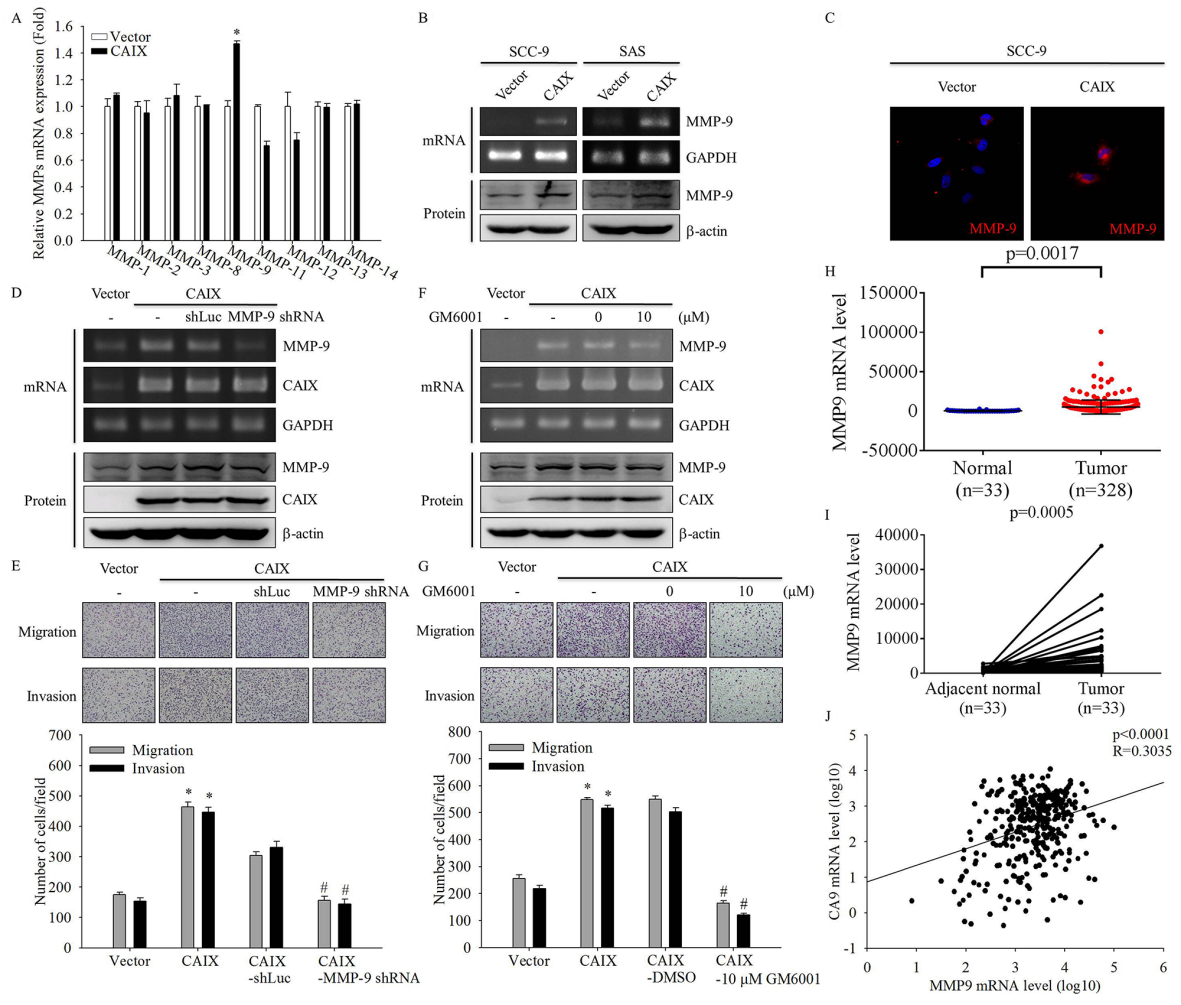
CAIX upregulates MMP-9 in oral cancer cells **(A)** The mRNA levels of MMPs in SCC-9 cells with or without CAIX expression were analyzed using quantitative real-time PCR. The data are presented from three independent experiments. **(B)** The mRNA and protein expressions of MMP-9 in SCC-9 and SAS cells were detected using RT-PCR (upper) and Western blotting (lower), respectively. **(C)** The expression of MMP-9 protein in SCC-9 cells was detected using ICC. **(D)** Knockdown of MMP-9 was confirmed by RT-PCR (upper) and Western blot analysis (lower) of cells treated with shRNA targeting MMP-9. Control cells were treated with shLuc. GAPDH and b-actin were used as loading controls. **(E)** Cells treated with shRNA targeting MMP-9 were analyzed using the Boyden chamber assay. (upper) Images were obtained using an inverted contrast light microscope under 100× magnification. (lower) The number of migrated and invaded cells per well was counted in three arbitrary visual fields. The results are expressed as mean ± SD of three replications. *p < 0.05 versus vector control; #p < 0.05 versus CAIX expression cells treated with shLuc. **(F)** CAIX-overexpressing SCC-9 cells were treated with 10 mM GM6001 for 24 h. RT-PCR (upper) and Western blotting (lower) were performed to detect the mRNA and protein levels of MMP-9 and CAIX. GAPDH and b-actin were used as loading controls. **(G)** Boyden chamber assay was used to examine the effects of an MMP-9 inhibitor (GM6001) on the migratory and invasive abilities of SCC-9 cells with CAIX expression. (upper) Images were obtained using an inverted contrast light microscope under 100× magnification. (lower) The number of migrated and invaded cells per well was counted in three arbitrary visual fields. The results are expressed mean ± SD of three replications. *p < 0.05 versus vector control; #p < 0.05 versus CAIX expression cells treated with DMSO. **(H)** Analyses of MMP-9 expression in normal oral tissues (n = 33) and oral tumors (n=328) in TCGA microarray data set. **(I)** Analyses of MMP-9 expression in 33 pairs of normal and tumor oral tissues in TCGA microarray data set. **(J)** Analyses of the correlation between CAIX and MMP-9 in normal and tumor oral tissues in TCGA microarray data set.

Because cell invasion and metastasis are often associated with the enhanced expression of MMPs [21-24], we examined the effect of CAIX on expression of MMP family by using real-time PCR. The data demonstrated that MMP-9 expression was markedly increased in CAIX-transfected SCC-9 cells (Figure 3A). Furthermore, we confirmed the mRNA and protein expression of MMP-9 through RT-PCR, Western blotting, and ICC analysis. The results showed that CAIX overexpression significantly induced MMP-9 mRNA and protein expression (Figure 3B and 3C). To address the role of MMP-9 in CAIX-induced cell migration and invasion, we examined the migratory and invasive abilities of CAIX-overexpressing SCC-9 cells treated with MMP-9 shRNA or the MMP inhibitor GM6001. MMP-9 expression in CAIX-overexpressing SCC-9 cells was effectively knocked-down by shRNA targeting MMP-9 and was inhibited by GM6001, as demonstrated by RT-PCR and Western blotting (Figure 3D and 3F). Suppression of MMP-9 expression by MMP-9 shRNA or GM6001 resulted in a significant reduction of the migration and invasion of CAIX-overexpressing SCC-9 cells (Figure 3E and 3G). These results demonstrated that CAIX induces cell migration and invasion through MMP-9 expression. We also analyzed the data on MMP-9 genes from TCGA data sets and found that MMP-9 mRNA expression was significantly higher in oral cancer tumor tissues than in matched adjacent normal tissues (Figure 3H and 3I). TCGA data sets revealed that the mRNA level of CAIX was significantly correlated with MMP-9 expression in oral cancer cells (r = 0.3035, p < 0.0001; Figure 3J). These results indicated that the increased expression of MMP-9 may mediate the increased cell migratory and invasive abilities associated with CAIX upregulation in oral cancer cells.

### FAK/Src and ERK signaling pathways were involved in CAIX-induced MMP-9 upregulation and cell migration and invasion

**Figure 4:**
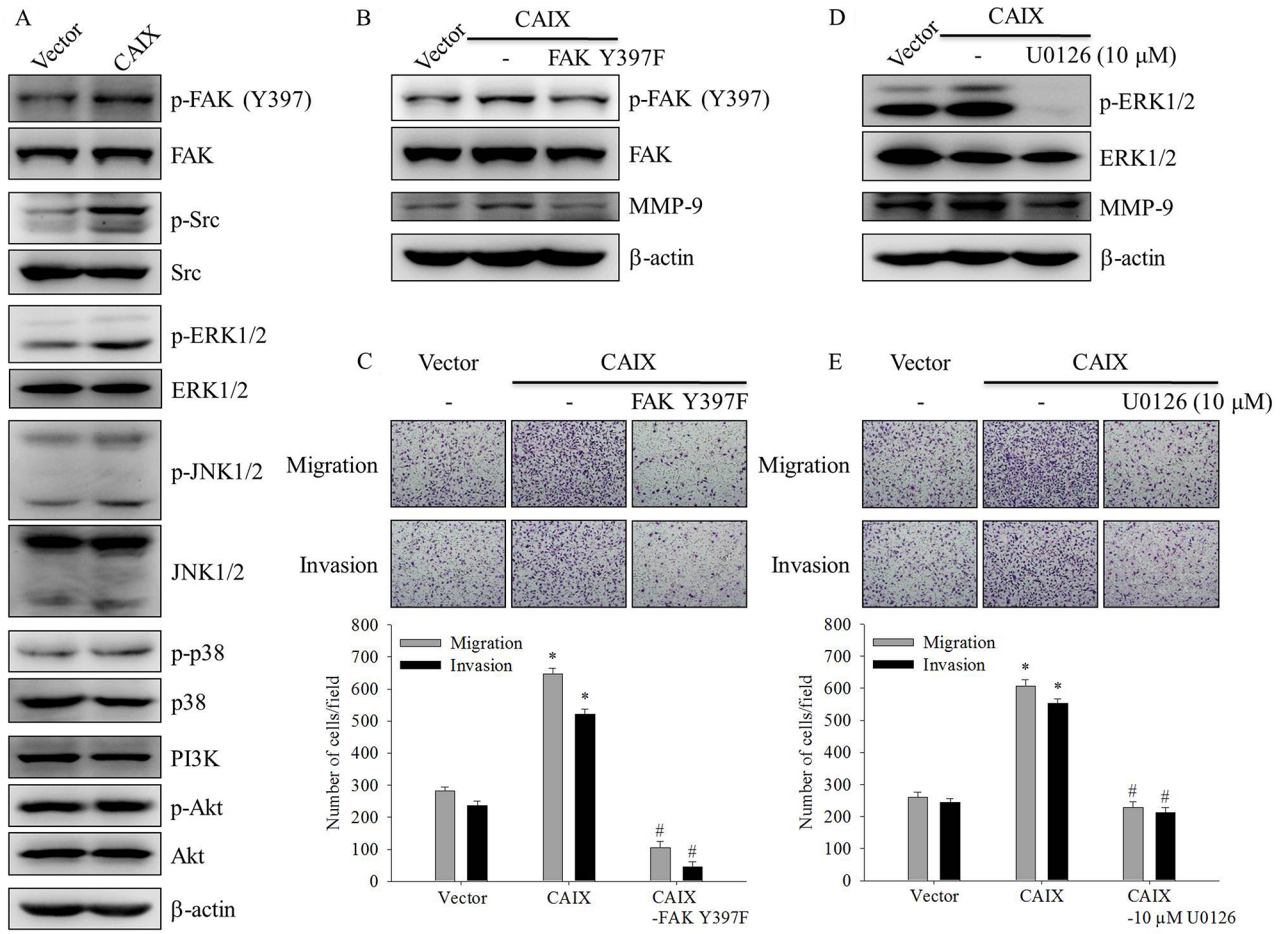
The FAK/Src and ERK signaling pathways are crucial for CAIX-induced MMP-9 upregulation and cell migration and invasion **(A)** The levels of total and phosphorylated FAK, steroid receptor coactivator (Src), ERK1/2, JNK1/2, p38, and Akt in SCC-9 cells with or without CAIX expression were determined using Western blot analyses of whole cell lysates. b-actin was used as a loading control. **(B)** SCC-9 cells with CAIX expression were treated with FAK Y397F for 24 h. Whole cell lysates were collected, and the expressions of FAK and MMP-9 were determined using Western blotting. b-actin was used as a loading control. **(C)** Cells treated with FAK Y397F for 24 h were analyzed using the Boyden chamber assay. (upper) Images were obtained using an inverted contrast light microscope under 100× magnification. (lower) The number of migrated and invaded cells per well was counted in three arbitrary visual fields. The results are expressed as mean ± SD of three replications. *p < 0.05 versus vector control; #p < 0.05 versus CAIX expression. **(D)** SCC-9 cells with CAIX expression were treated with 10 mM U0126 for 24 h. Western blotting was used to examine the effects of U0126 on ERK and MMP-9 expression. b-actin was used as a loading control. **(E)** Cells treated with 10 mM U0126 for 24 h were seeded into the Boyden chamber and were incubated at 37°C for 16 h. (upper) Images were obtained using an inverted contrast light microscope under 100× magnification. (lower) The number of migrated and invaded cells per well was counted in three arbitrary visual fields. The results are expressed as mean ± SD of three replications. *p < 0.05 versus vector control; #p < 0.05 versus CAIX expression.

To elucidate the signaling pathways involved in CAIX-induced MMP-9 upregulation and cell migration and invasion, we detected the activation status of signaling molecules in CAIX-overexpressing SCC-9 cells. The results showed that the phosphorylation of FAK, Src, and ERK1/2 increased in CAIX-overexpressing SCC-9 cells (Figure 4A). To address the functional roles of FAK and ERK1/2 in MMP-9 expression and the invasive and migratory phenotypes of CAIX-overexpressing SCC-9 cells, an FAK dominant negative mutant (FAK Y397F) and a specific inhibitor of MEK (U0126) were used. The knockdown of FAK expression by the treatment of SCC-9 cells with FAK Y397F decreased the levels of active FAK and MMP-9 expression (Figure 4B). Moreover, FAK Y397F treatment significantly inhibited the migration and invasion of CAIX-overexpressing SCC-9 cells (Figure 4C). U0126 treatment significantly suppressed phosphorylated ERK1/2 and MMP-9 expression in CAIX-overexpressing SCC-9 cells (Figure 4D). Moreover, the treatment of SCC-9 cells with U0126 markedly attenuated CAIX-induced cell migration and invasion (Figure 4E). These results indicated that the activation of the FAK/Src and ERK signaling pathways is required for CAIX-induced MMP-9 upregulation and cell migration and invasion.

### Transcription factor NF-κB and AP-1 were involved in CAIX-induced MMP-9 upregulation

**Figure 5:**
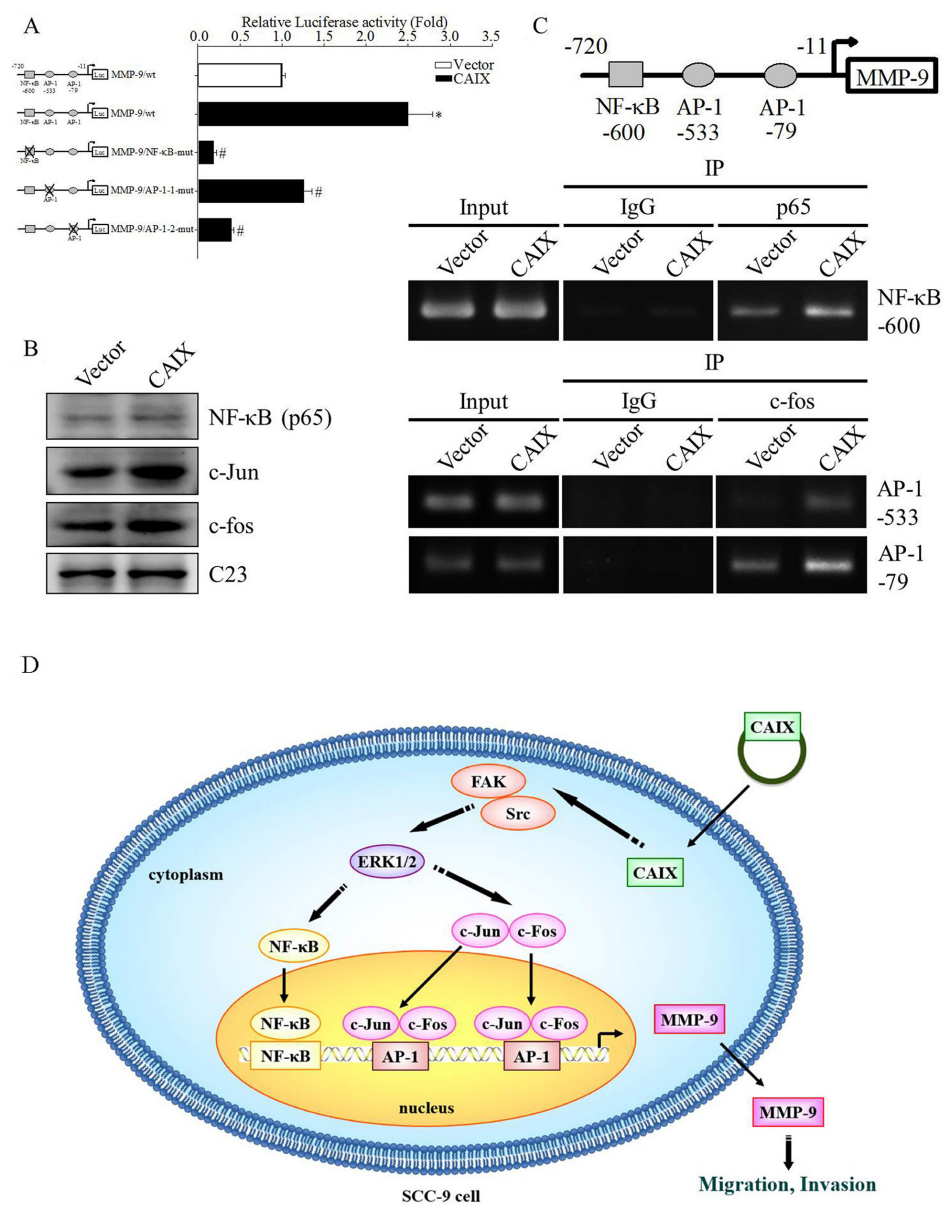
Transcription factors NF-κB and AP-1 are essential for CAIX-dependent MMP-9 expression **(A)** The reporter constructs of full-length or mutant (the mutation of the NF-κB or AP-1 binding elements) MMP-9 promoter were cloned in the pGL3 luciferase reporter vector. The luciferase reporter assay was used to analyze the effects of the mutation of the NF-κB or AP-1 binding elements on the MMP-9 promoter activity of cell extracts. The results are expressed as mean ± SD of three replications. *p < 0.05 versus vector control; #p < 0.05 versus CAIX expression. The data are presented from three independent experiments. **(B)** The levels of NF-κB, c-Jun, and c-Fos in SCC-9 cells with or without CAIX expression were determined through Western blot analysis of the nuclear fraction. C23 was used as a loading control. **(C)** ChIP assay was performed to determine the binding of NF-κB or AP-1 to the MMP-9 promoter. IgG was the negative control, and it was input as the positive control. **(D)** Proposed mechanism by which CAIX induces oral cancer cell motility.

Previous studies have suggested that several transcription factors are involved in the regulation of human MMP-9 expression [25, 26]. To determine which transcription factor is responsible for CAIX-induced MMP-9 expression, the full-length construct and a mutation of NF-κB or AP-1 binding elements of the MMP-9 gene promoter were cloned into the pGL3 luciferase reporter vector, and a luciferase reporter assay was performed to measure the transcriptional activity of the MMP-9 promoter in CAIX-overexpressing SCC-9 cells. The results revealed that the transcriptional activity of the MMP-9 promoter was 2.5-fold higher in CAIX-overexpressing SCC-9 cells than in control cells, whereas the mutation of NF-κB or AP-1 binding elements dramatically reduced CAIX-dependent MMP-9 promoter activity (Figure 5A). The results indicated that the NF-κB or AP-1 binding elements are required for the CAIX-dependent activation of the MMP-9 promoter. Moreover, we used Western blotting to investigate the expression of the NF-κB (p65), c-fos, and c-jun transcription factors in the nuclear. The results revealed that the nuclear levels of NF-κB (p65), c-fos, and c-jun were significantly increased in CAIX-overexpressing SCC-9 cells (Figure 5B). Furthermore, the results of a chromatin immunoprecipitation (ChIP) assay demonstrated that the binding of NF-κB/AP-1 to the MMP-9 promoter was enhanced by CAIX overexpression in SCC-9 cells (Figure 5C). These results suggested that NF-κB and AP-1 are essential for CAIX-dependent MMP-9 activation.

## DISCUSSION

Metastasis is responsible for the majority of cancer-related deaths [27, 28]. Most cancers are solid tumors that are prone to occur following hypoxia. CAIX is a hypoxia-induced transmembrane protein that is highly expressed in various types of cancer. A previous study reported that CAIX promotes tumor-associated cell migration and invasion in the human cervical carcinoma cell line C33A [29]. Therefore, CAIX may play an important role in cancer metastasis. In this study, we hypothesized that CAIX promotes the metastasis of oral cancer cells. Consequently, we found that CAIX increased the migration and invasion of oral cancer cells. This study was the first to demonstrate that CAIX can promote oral cancer cell migration and invasion through MMP-9 expression. We also showed that the activated FAK/Src and ERK signaling pathways and NF-κB/AP-1 are crucial for CAIX-dependent MMP-9 expression and cell migratory and invasive abilities (Figure 5D).

The proteinase-mediated degradation of the ECM and basement membrane is one of the crucial steps in cancer metastasis. In human cancer, MMPs are conventionally correlated with matrix remodeling, in particular with cancer invasion and angiogenesis [30]. The activation of MMPs is precisely regulated at three levels: gene transcription, the post-transcriptional activation of zymogens, and the endogenous expression of the tissue inhibitors of metalloproteinases [31]. Transcription-level regulation is considered to be the key step in the regulation of MMPs [32-35]. In this study, we found that CAIX overexpression induced MMP-9 expression and cell migration and invasion in human oral cancer cells. The present finding is consistent with previous reports that MMP-9 overexpression in OSCC cells increased cell migration and invasion *in vitro* and tumor growth and lymph node metastasis *in vivo* [33, 35-39]. In addition, the inhibition of CAIX-enhanced MMP-9 protein expression through treatment with shRNA or GM6001 significantly suppressed CAIX-induced cell migration and invasion. Therefore, MMP-9 may be the CAIX-responsive mediator that causes the degradation of the ECM, which may lead to subsequent cancer metastasis.

AP-1 and NF-κB are two key transcription factors involved in the regulation of MMP-9 gene expression [40]. In this study, the luciferase reporter assay and the mutation analysis of the promoter revealed that the major target of the MMP-9 promoter was AP-1 and NF-κB, which regulate MMP-9 transcriptional activity. AP-1 is composed of proteins belonging to the c-Jun and c-Fos families [41]. Our results showed that CAIX increased nuclear NF-κB, c-Jun, and c-Fos protein expression. The ChIP assay suggested that AP-1 and NF-κB are responsible for CAIX-induced MMP-9 expression. AP-1 and NF-κB are modulated by protein kinases such as mitogen-activated protein kinases. In our experiments, CAIX overexpression increased OSCC migration through the phosphorylation of ERK1/2 without affecting the pathways involving p38 and JNK. U0126 treatment reduced CAIX-mediated MMP-9 expression and cell migration and invasion. This finding is consistent with previous reports that the ERK1/2 signaling pathway plays an important role in oral cancer cell migration and invasion [42-44]. Moreover, previous studies have shown that FAK plays a critical role in contact formation between the ECM and cytoskeleton, and FAK has been linked to cancer cell migration, invasion, survival, and proliferation [45-47]. In this study, we demonstrated that CAIX increased the phosphorylation of tyrosine 397 in FAK and Src. Furthermore, the FAK mutant FAK Y397F antagonized CAIX-mediated MMP-9 expression and cell migration and invasion abilities. This finding suggests that FAK activation is an obligatory event in the CAIX-induced migration and invasion of oral cancer cells. Future studies should address the mechanism by which CAIX regulates FAK activation in OSCC.

In summary, CAIX induces oral cancer cell migration and invasion by increasing MMP-9 expression, which is mediated through the phosphorylation of protein kinases (FAK/Src and ERK1/2) and the activation of AP-1 and NF-κB transcription factors. The present observations suggested that CAIX has a novel function in promoting cancer cell migration and invasion and may be a therapeutic target for oral cancer.

## MATERIALS AND METHODS

### Cell lines and cell culture

SCC-9 and SAS cell lines were obtained from ATCC (Manassas, VA, USA) and the JCRB Cell Bank (Osaka, Japan), respectively. Both cell lines were cultured in Dulbecco’s modified Eagle’s medium, accompanied by a nutrient mixture comprising F-12 Ham’s medium, as previously described [48].

### Establishment of stable SCC-9 and SAS cell lines overexpressing CAIX

The cDNA of CAIX was amplified using a polymerase chain reaction (PCR) and it was cloned into the pcDNA3.0 vector. SCC-9 and SAS cells were transfected with the pcDNA3.0-CAIX or pcDNA3.0 vector by using Lipofectamine™ 2000 (Invitrogen, Carlsbad, CA, USA) and were then treated with G418. After G418 selection for 3 weeks, only stable clones with CAIX overexpression or the CAIX overexpression vector were obtained.

### Reverse transcription-PCR and quantitative real-time PCR

Total RNAs were isolated from SCC-9 and SAS cells by using the Total RNA Mini Kit (Geneaid Biotech Ltd, Sijhih City, Taiwan), and cDNAs were reverse transcribed from isolated total RNA (5 mg) by using the High Capacity cDNA Reverse Transcription Kit (Applied Biosystems, Foster City, CA, USA). A semiquantitative reverse transcription (RT)-PCR and real time PCR were performed as previously described [48].

### Western blotting

Total cell lysates were separated on a 10% sodium dodecyl sulfate (SDS)-polyacrylamide gel and transferred onto a nitrocellulose membrane. The blot was subsequently incubated with 5% bovine serum albumin (BSA) (Sigma Chemical Co., St. Louis, MO, USA) in Tris-buffered saline (11 mM Tris, 154 mM NaCl, pH 7.4) containing 0.1% Tween-20 for 1 h to block nonspecific binding, and the blot was performed according to the methods described by Hsin et al [39].

### Immunocytochemistry

Cells were seeded into 8-well chamber slides overnight and then fixed with 4% paraformaldehyde for 20 min. Subsequently, cells were permeabilized with 0.5% Triton X-100 diluted in phosphate-buffered saline (PBS) for 10 min, blocked with 1% BSA for 1 h, and incubated with primary antibodies at 4°C overnight and then with fluorescent secondary antibodies at room temperature for 1 h. Cells were counterstained with 4’,6-diamidino-2-phenylindole (DAPI) and mounted on glass slides and were then analyzed using confocal microscopy.

### MTT assay

An MTT colorimetric assay was performed to determine cell proliferation [39]. Cells at a density of 1 × 10^4^ cells/well were seeded into 24-well plates at 37°C for 1-3 days. Thereafter, cells were incubated with MTT (5 mg/mL; Sigma Chemical Co., St. Louis, MO, USA) for 4 h. After solubilization with isopropanol, the viable cell number per well, which is directly proportional to formazan production, was measured spectrophotometrically at 563 nm (Hitachi U-1900 spectrophotometer; Hitachi High-Technologies Corporation, Tokyo, Japan).

### Cell cycle analysis (flow cytometry)

Cells were harvested with trypsin–ethylenediaminetetraacetic acid (EDTA), washed twice with PBS, and fixed in 70% ethanol at −20°C overnight. The fixed cells were pelleted, stained with propidium iodide in darkness for 20 min at room temperature, and analyzed using flow cytometry (FACS Calibular; BD Biosciences, Bedford, MA, USA).

### Wound healing assay

Cells were grown in 6-cm culture dishes until confluence. Wounds were generated by scratching the cell monolayer with a sterile pipette tip. Closures of these wounds were documented photographically after 0, 12, 24, and 48 h under an inverted phase-contrast microscope (Olympus Corporation, Tokyo, Japan).

### Boyden chamber assay

Cell invasion and migration assays were performed according to the methods described by Ho et al. [49].

### Luciferase reporter assay

SCC-9 cells were transiently transfected with the pGL3-MMP-9 promoter luciferase reporter plasmid and the b-galactosidase plasmid by using Lipofectamine™ 2000 (Invitrogen). After incubation for 24 h, these cells were lysed using reporter lysis buffer (Promega, Madison, WI, USA), and after incubation in luciferase assay reagent (Promega, Madison, WI, USA), the lysate was assayed for luciferase activity on a luminometer. Luciferase activity was normalized to the transfection efficiency monitored using the cotransfected b-galactosidase expression vector.

### Chromatin immunoprecipitation assay

Cells were washed twice with ice-cold PBS and were then treated with 1% formaldehyde to cross-link protein–DNA and protein–protein complexes. After cells were lysed using lysis buffer (1% SDS, 10 mM EDTA, 50 mM Tris–HCl; pH 8.1, protease inhibitor cocktail tablets), chromatin was fragmented using sonication and was immunoprecipitated using rabbit polyclonal antibody or rabbit immunoglobulin G antibody as the negative control as previously described [49].

### Statistical analyses

Values are expressed as mean ± SD. The statistical significances of differences obtained in this study were calculated using Student’s t-tests. Oneway analysis of variance (ANOVA) test were used for analysing three or more groups. Survival curves from The Cancer Genome Atlas (TCGA) dataset were analyzed using the Kaplan–Meier method and were compared using the log-rank test. A difference with a p value of < 0.05 was considered to be statistically significant.

## Abbreviations

CAIX: carbonic anhydrase IX
ECM: extracellular matrix
Erk1/2: extracellular signal-regulated kinase 1/2
FAK: focal adhesion kinase (FAK)
HIF-1: hypoxia-inducible factor-1
MMP-9: matrix metalloproteinase-9
NF-κB: nuclear factor-κB
OSCC: oral squamous cell carcinoma
Src: steroid receptor coactivator
